# Reversibility of link prediction and its application to epidemic mitigation

**DOI:** 10.1038/s41598-022-25023-6

**Published:** 2022-12-03

**Authors:** Sadegh Sulaimany, Aso Mafakheri

**Affiliations:** Social and Biological Network Analysis Laboratory (SBNA), Department of Computer Engineering, University of Kurdistan, Sanandaj, Iran

**Keywords:** Computer science, Computational models, Network topology

## Abstract

Current link prediction strategies are about finding new probable strong relations to establish or weak ones to remove. An interesting strategy is utilizing link prediction to prioritize the edges in the network and finding newly probable established relations. In this paper we will introduce and explain RLP, reverse link prediction, as a new paradigm, and use popular basic scoring methods including CN, JC, AA, RA, and PA, as its core to examine. The test cases are nine datasets. Half of them are contact networks in different levels from personal contact to aviation, and another half is for covering different test situations. After reviewing the edge removal based epidemic mitigation methods, we show that RLP can be used to decrease the epidemics spreading speed as a general method with various link prediction algorithms, and here in this paper, preferential attachment (PA) has the best results overall. But the results heavily depend on the nature of the examined networks: regular, scale-free or small-world. We also propose an easy to understand criteria, path count, for comparing the efficacy of epidemics mitigation methods. RLP can be extended to use other link prediction scoring methods in various types of graphs as well.

## Introduction

Link prediction as an important graph mining task is receiving increasing attention from several viewpoints. First, improving the accuracy and extendibility of current methods. For instance, proposing stronger algorithms for link prediction in sparse networks. Second, application of link prediction to new areas in addition to social, biological, scientific, etc. Third, novel strategies for utilization of link prediction algorithms. For example, finding most probable weak links to remove from the network, or simultaneous prediction of establishing and disappearing relations in a network instead of just finding new connections. Accordingly, literature^1^ proposed four strategies for link prediction: Positive, Negative, Mixed and Reverse. While positive link prediction (PLP) tries to find the new probable relations, negative link prediction (NLP) is going to discover the weak connections that will disappear in new future. When implementing both PLP and NLP at the same time, we use mixed link prediction (MLP)^2^. Another interesting link prediction strategy that can be used to inform new recently established links is reverse link prediction (RLP).

In this paper we are going to explain and investigate RLP strategy, and one of its applications for decreasing epidemics propagation speed. Therefore, our contribution is threefold: first, explaining a new link prediction strategy, RLP, second, proposing a novel method for epidemics mitigation as the application of RLP, and finally, offering a new simple and easy to calculate criteria for measuring the efficacy of computational epidemic mitigation methods.

Structure of the paper is as follows. The second section of this paper explains the reverse link prediction idea. We will also review edge removal based epidemic control methods plus introducing a new evaluation criterion in the next section titled edge removal-based epidemics control. The material and method section is explaining the three-step proposed method with assessment metrics after describing nine examined networks. Results and their discussion will be presented in subsequent sections. The last section, summarizes the research and introduces potential future works, as well.

## Reverse link prediction

Basically, link prediction is a mechanism that ranks the absent relations in a graph for the purpose of finding the most probable ones as missing or for establishing. This problem can be formulated in its simplest form as follows: Given a snapshot of a graph *G(V,E)* at time *t*_*0*_, where *V* is the set of nodes and *E* is the set of links, which new edges are likely to be established among vertices at time *t*_1_ (*t*_1_ > *t*_0_)?^3^. However, link prediction problem is not necessarily depended on time and temporal state of the network. It has been used for different networks such as social and biological networks, and there are several published surveys about it^4–7^. Even though quite often the methods of link prediction methods are focusing on predicting the future of the network by adding new links, a few papers predict the most likely links to be cut from the network^2,8^. Similar to literature^1^, we insert the PLP, NLP and MLP approaches into a category named forward link prediction that tries to predict establishment or removal of the relations.

**Figure 1:**
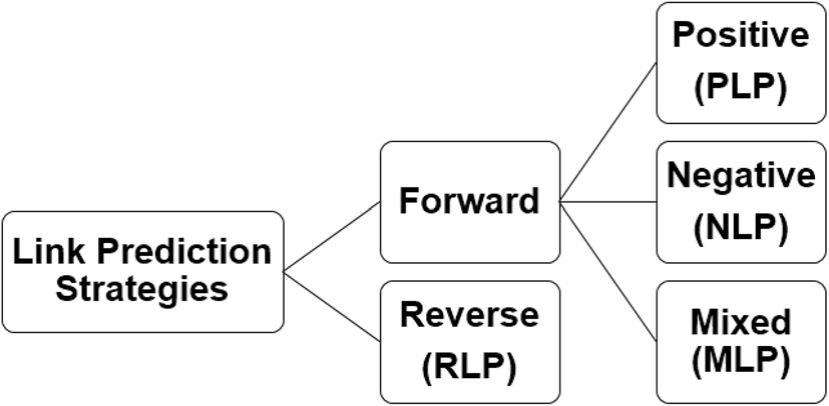
A extensive categorization of link prediction strategies; dividing it into two main categories: forward and reverse. The forward approach itself can be positive, negative or mixed as well.

However, for some applications we are interested in finding new established or redundant edges in the graph. For example, for epidemics mitigation, we are eager to discover the most redundant relations that can be controlled or blocked with the minimum side effects on the network interactions. In other words, here we are going to prioritize the current links without adding or removing relations from the network. Another view to this idea is finding the most currently established links. Blocking such edges, for example in epidemics control, will force the epidemics to find longer path to flow among the network and so delays the propagation speed. We will refer to this type of link prediction as *Reverse Link Prediction (RLP)* and will explain it in the material and methods section of the paper. Based on these concepts, new categorization of link prediction strategies is shown in Fig. 1.

Besides, suitable ranking formula is needed to be used in RLP strategy in order to find or prioritize the best edges. We use some basic algorithms, node neighborhood similarity-based ones, as the core of RLP because of its short running time, low computational complexity and good accuracy, that make it appropriate and effective choice for evaluating new ideas^9,10^. There are five common node neighborhoods similarity-based link prediction methods^5^: Jaccard (JC), Common Neighbors (CN), Adamic & Adar (AA), Resource Allocation (RA) and Preferential Attachments (PA). Their distinction returns to their prediction score function. Score functions for the methods are defined as described in for a link with *x* and *y* nodes at its ends (Table 1). $\Gamma (x)$ is the set of the neighbors of node *x* and $\left| {\Gamma (x)} \right|$ is the set size, number of *x* neighbors.

**Table 1:** Score functions used by node neighborhood similarity-based link prediction methods^5^ to rank an edge confined to *x* and *y* nodes in RLP strategy.

Link prediction methods	Score function
Common neighbors (CN)	$\left| {\Gamma (x)} \right. \cap \left. {\Gamma (y)} \right|$
Jaccard (JC)	$\frac{{\left| {\Gamma (x)} \right. \cap \left. {\Gamma (y)} \right|}}{{\left| {\Gamma (x)} \right. \cup \left. {\Gamma (y)} \right|}}$
Preferential attachments (PA)	$\left| {\Gamma (x)} \right|.\left| {\Gamma (y)} \right|$
Resource allocation (RA)	$\sum\limits_{s \in \Gamma (x) \cap \Gamma (y)} {\frac{1}{{\left| {\Gamma (s)} \right|}}}$
Adamic & Adar (AA)	$\sum\limits_{s \in \Gamma (x) \cap \Gamma (y)} {\frac{1}{{\log_{2} (\left| {\Gamma (s)} \right|)}}}$

General pseudocode for RLP strategy is as Algorithm 1. For the simplicity, the modeling graph has supposed to be simple without direction or weight for the edges. Also, algorithm uses the score functions at Table 1. This algorithm gets a Boolean adjacency matrix of epidemics network for example, and returns an existing edge (*x,y*) with the highest rank, TopRank. TopRank will be used to remove or control the most appropriate edge. The algorithm only does the calculations on half of the matrix, as the matrix is symmetric, not directional. We will use this algorithm repeatedly to remove a desired number of top edges from the network. Notably, the only difference of this algorithm with PLP is in line 5, where we check the matrix entries against 1 value to rank the existing edges, while we may check the 0 entries of the matrix for PLP in order to rank the non-existent links. 
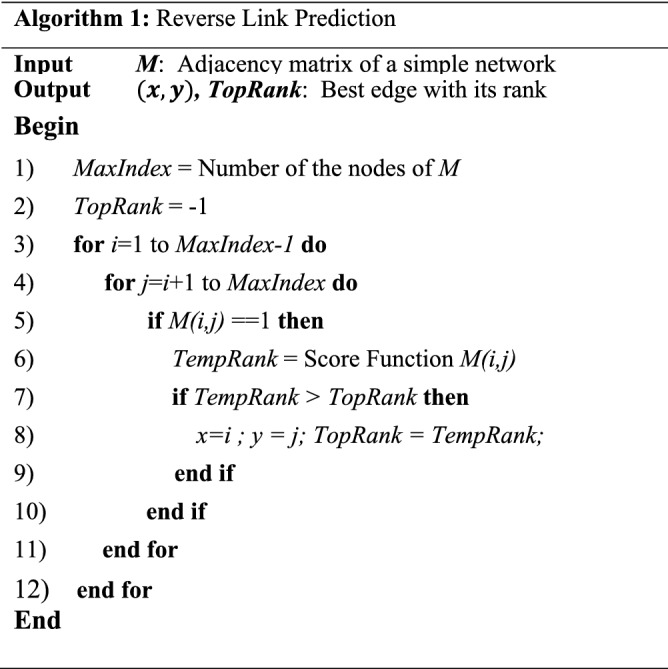


## Edge removal-based epidemics control

The control of disease spreading is very important to avoid potentially fatal effects. Disease spreading mainly occurred through human contact and traveling networks. Swine influenza, SARS, Ebola, Zika and recent COVID-19 outbreak are some of the most important cases. However, minimizing the spread of unpleasant issues is not just devoted to diseases. Misinformation in social networks, virus propagation in computer networks and several related examples maybe considered as well.

Many computational studies have proposed removing or quarantining highly connected nodes from the epidemic’s networks for containment of the epidemic spreading^11^ while some disease epidemics studies show the usefulness of relation or edge removal methods^12^. Some reasons are that it is not always possible to remove the node from the disease networks, but removing or controlling the links is easier and more applicable. For example, stopping or closing the airport to reduce the epidemic spreading is very expensive, or vaccinating critical nodes in the disease network cannot probably decrease the speed of the epidemic spreading for all the cases because there is not on time vaccine available for every disease; such as Ebola, hepatitis C, HIV, and emerging influenza strains like new Coronavirus versions.

Various edge control or removal strategies have been proposed to epidemic mitigation. Finding the best connection to be removed from the network in order to minimize the epidemic mitigation is an NP-hard problem^13^. Reference^14^ has presented a categorization of the methods based on “preventive” or “reactive” approaches. While preventive approach attempts to prioritize the edges to control or monitor before any disease outbreak and relies only on the network structure, reactive approach takes the information about initial disease state into account to decide which links to remove or control after starting and during the epidemics. There are several important parameters for epidemic mitigation solutions:

- Preventive or reactive perspective of the solution.
- Method for selecting the most appropriate edges to monitor or remove.
- The spreading models.
- Starting nodes of spreading, initial infected nodes.
- Stop population or edge removal stop time.
- The evaluation method.

In Table 2 we have enumerated the above parameters for available edge removal based epidemic mitigation papers. The application area of the papers is diverse for example form disease spreading^12,15–17^ to spam, worm, virus, rumor, error or even information epidemics in computer networks^18,19^. Also, their granularity level of the epidemics is different from personal contacts to flight traveling networks.

**Table 2:** Comparison of the edge removal based epidemic mitigation papers according to six important parameters.

References	Approach	Method	Spreading model	Start nodes	Edge removal stop time	Evaluation metric
^12^	Preventive	Edge betweenness centrality and Jaccard coefficient	SI	Any node	Infecting half of the nodes	Average number of time steps for infecting half of the nodes
^19^	Preventive	Dual problem to the influence maximization problem	SIR	Any node	Until blocking a limited number of links	Average and maximum of influence degrees of all the nodes should be the minimum
^20^	Reactive	Using use a linear control model	SEIR	Multi-group	Reduce certain spread fractions to 0	Use the total of the spread performance statistics
^18^	Reactive	Clustering based link removal	SIS	Any node	The virus be quarantined in one or more clusters	Size of clusters and epidemic threshold
^17^	Reactive	Relaxed convex optimization protocol	SIRS	Any node	After removing a constraint number of links	Minimizing extreme eigenvalue (${\uplambda }_{m}$) of the network
^21^	Reactive	Minimizing the number of initially susceptible nodes via QCQP formulation	SI	Randomly select initial infected nodes	After removing the K links according to problems limitations	Compare fraction of susceptible nodes saved from infection
^16^	Preventive	Focusing on the best spreaders in a network	SI	Networks core nodes	All the links	Decrease in the extreme eigenvalue (${\uplambda }_{m}$) of the network
^22^	Preventive	Targeted cutting of edges with the largest edge betweenness centrality	SIS	0.1 of randomly selected nodes	Until bringing the epidemic into a steady state	Decreasing the extreme eigenvalue or ${\uplambda }_{m}$
^15^	Reactive	A set of local strategies for social distancing, based on community structure	SPIR	Any node	Until no epidemics remains	Measuring the fraction of vertices that become infected and recover
^23^	Reactive	Mixed integer linear programs	SI and SIR	Any node	Time to infect half of the susceptible nodes	Minimize the number of connections or paths between susceptible and infected nodes
^24^	Reactive	Link betweenness centrality and random method	SIR	Any node	Testing different precents of network links from 5 to 95	Size of largest connected component

Reviewing the proposed methods of edge removal for decreasing the spread of epidemics shows that there is not a general and easy to understand and implement method. But the evaluation criteria are common for many of the studies; decreasing the extreme eigenvalue as much as possible for the related network^16,17,22^. Simply, the extreme eigenvalue of a network is a mathematical parameter that can be affected by the removal of the links from the network, and when it becomes as low as possible, the epidemics speed will be in its lowest rate^25^.

Evidently, there are some paper considering the edge removal effects on the network robustness. One application of such researches is preventing or decreasing the epidemic. Some other names of the problem are: critical or influential edge identification^26,27^. Major limitation of these researches is dependency on propagation modeling such as SIR that led to time complexity and uncertainty and lack of generalization. Reference^28^ provides a review of the current related literature, and paper^29^ extends the survey and comparison to weighted networks as well. Based on their findings, binary edge betweenness centrality has the best results in finding most effective relations independent from network relation weights. Therefore, this is the common algorithms that we can compare with our method.

Simply, edge betweenness centrality (hereafter, EB) is the most commonly used measure of a link’s importance in a network and has been widely used to find the appropriate connection to control or remove in order to mitigate the epidemic spreading^12,14–16,28^. It can be computed for a specified link or relation by fraction of the numbers of all shortest paths go through it given by^14^:

$$c(e) = \sum\limits_{(i,j)}^{{}} {\frac{\sigma (i,j|e)}{{\sigma (i,j)}}},$$

where $\frac{\sigma (i,j|e)}{{\sigma (i,j)}}$ is the fraction of the shortest paths between nodes *i* and *j* passing over link *e.*

## Materials and methods

### Investigated networks

We use several networks, disease and non-disease related ones, to test our idea. Disease related networks are ranged from human contact (Primary school proximity and Infect-Dublin) to road (Minnesota Road) and flight networks (Global Airline Route). Moreover, to cover the different conditions of network topologies, we investigated the Human proteins (Figeys), Email Network, Netscience, collaboration network between Jazz musicians and US power grid network as well.

Overall statistics and primary attributes of the provided networks is as Table 3. Network density, fraction of the potential connections in the network that are actually exists, varies from 0.0009 for Minnesota road to 0.28 for Primary school proximity. Also, Minnesota road demonstrate distinct properties for network diameter, density, average clustering coefficient (density of the relations between the neighbors of a node), and average shortest path length. The reason is that the Minnesota roads is based on real road topology that is similar to tree than graph structure.

**Table 3:** Utilized datasets to test the efficiency of RLP in epidemics containment.

Network	Node count	Edge count	Average degree	Maximum degree	Density	Diameter	Average clustering coefficient	Average shortest path length
Airline route	3397	*19,230*	11.32175	*248*	0.00333	13	0.48834	4.10324
Minnesota road	2640	3302	**2.50152**	**5**	**0.00095**	*99*	**0.01597**	*35.34908*
Primary-school-proximity	242	8317	*68.73554*	134	*0.28521*	**3**	0.52554	**1.73245**
Infect-Dublin	410	2765	13.4878	50	0.03298	9	0.45582	3.63085
Netscience	379	**914**	4.82322	34	0.01276	17	*0.74123*	6.04187
US power grid	*4941*	6594	2.6691	19	0.00054	46	0.0801	18.98919
Human proteins (Figeys)	1226	2410	3.93148	34	0.00321	17	0.06751	5.92896
Jazz	**198**	2742	27.69697	100	0.14059	6	0.61745	2.23504
Email-Univ	1133	5451	9.62224	71	0.0085	8	0.22018	3.60603

Minimum value for each property, column, has been bolded, and maximum value for each property has been italics*.*

**Figure 2:**
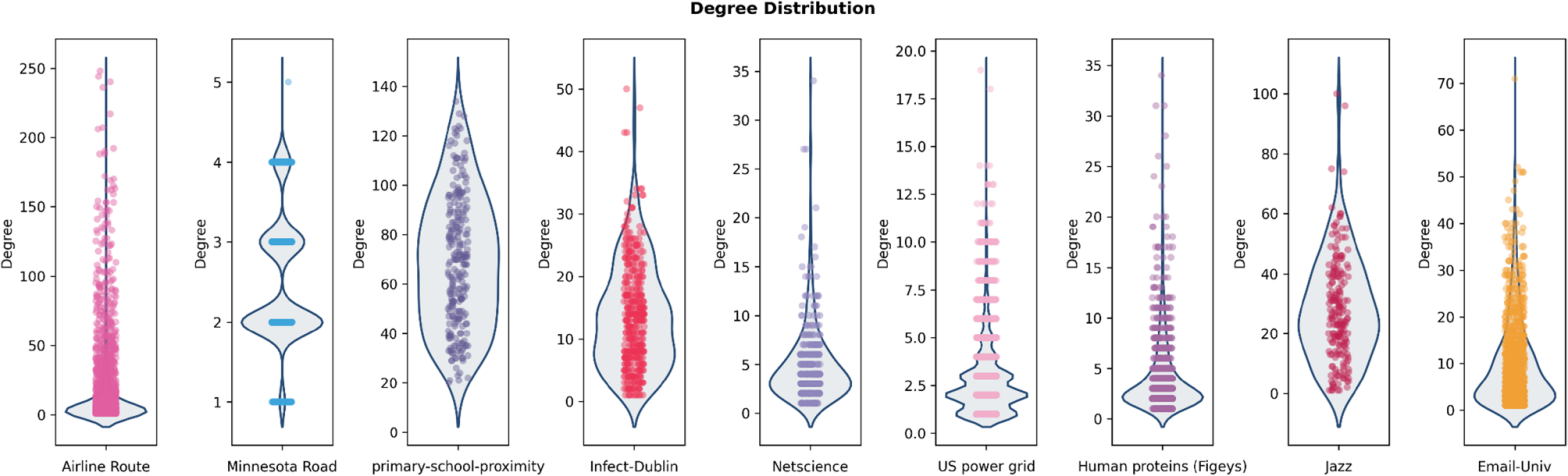
Violin plot of the degree distribution of probed datasets make the visual comparison easier.

**Figure 3:**
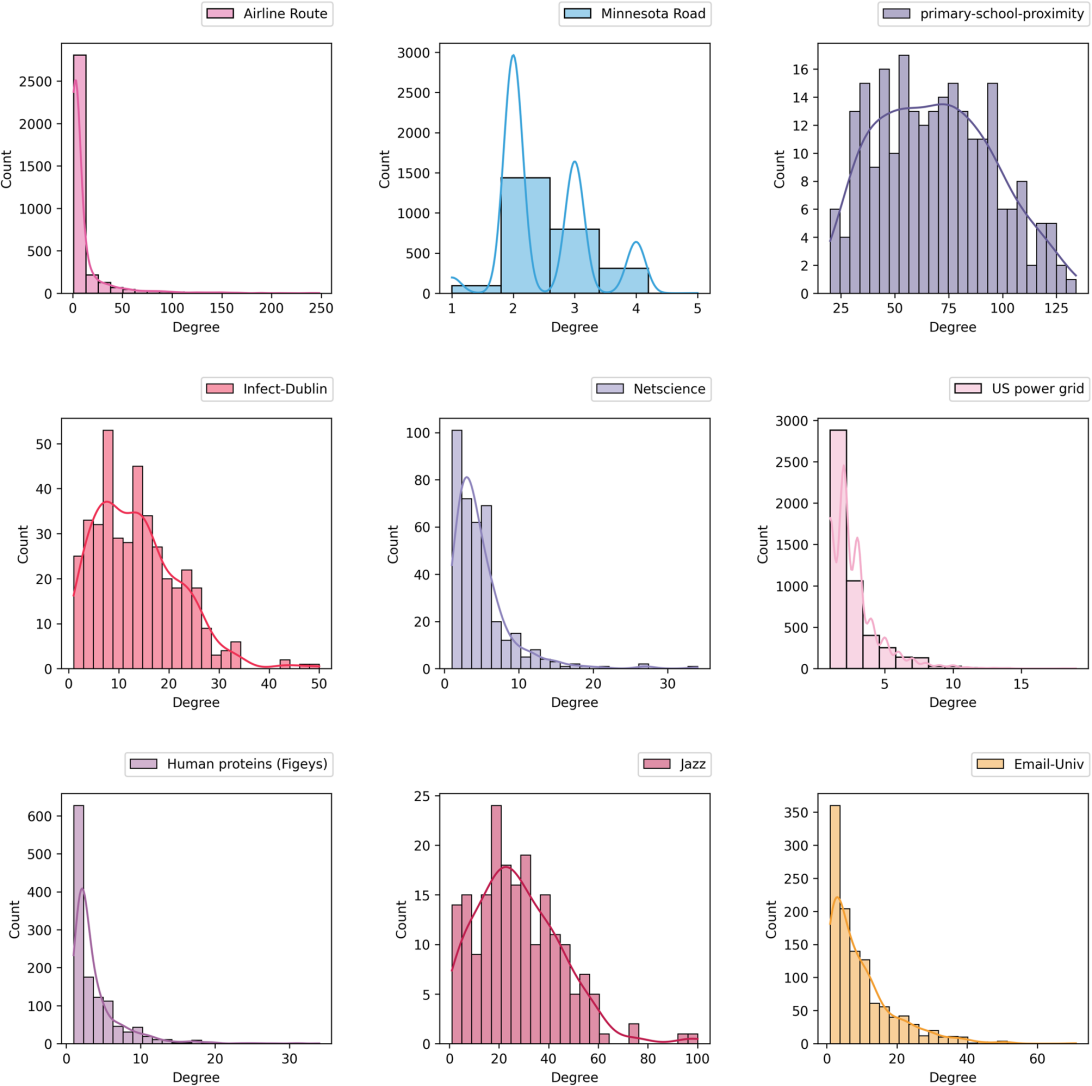
Degree distribution of the examined networks.

Nevertheless, degree distribution of the datasets gives more information about the properties of the examined networks (Figs. 2, 3). While Airline route, US Power grid, Human proteins and Email-univ charts are analogous to power-law distribution, Primary school proximity is more similar to normal distribution than others.

### Research workflow

**Figure 4:**
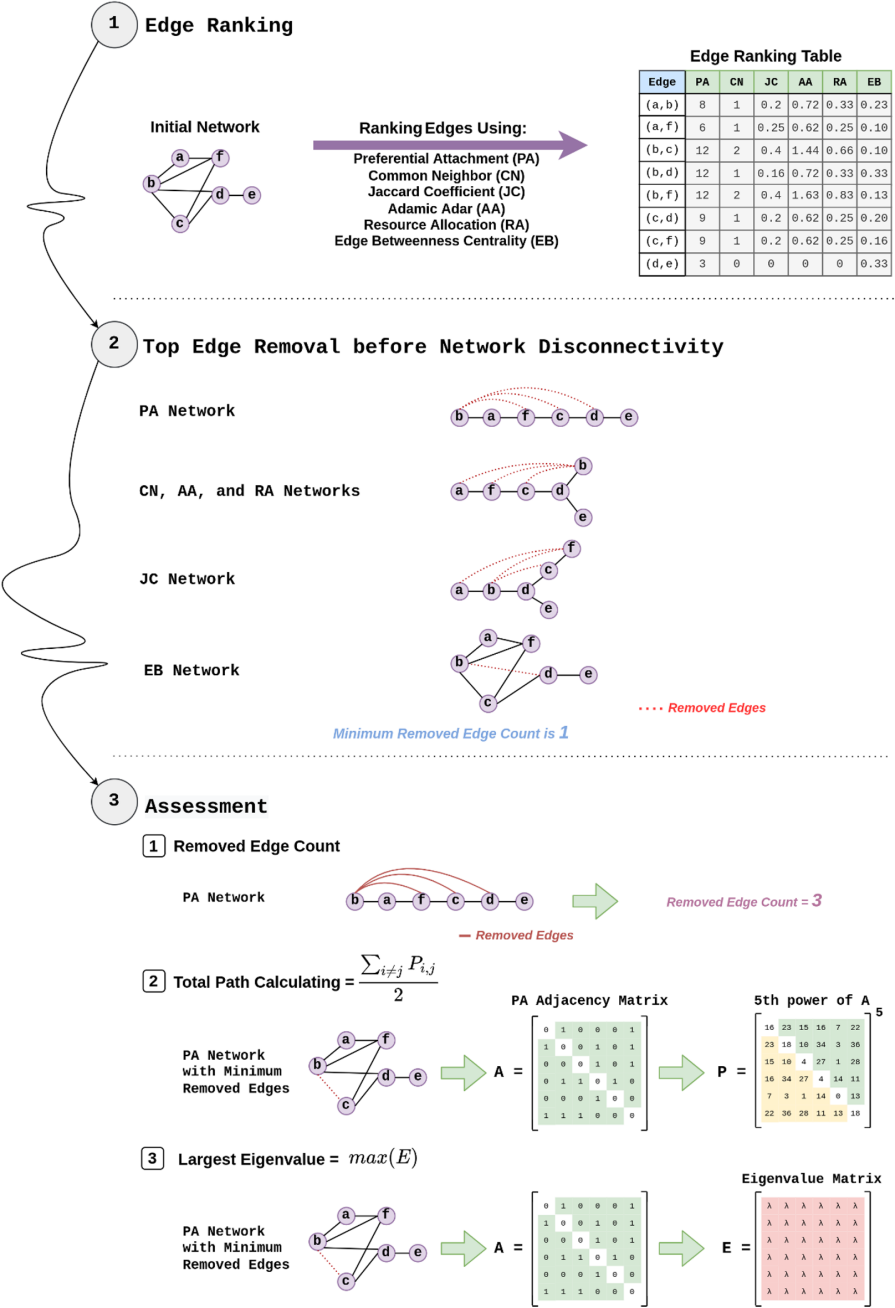
Overall research process to find the best epidemic mitigation score function used in RLP strategy.

Most edge control strategies noted in Table 2 are so diverse and different, and some of them are limited to special conditions. We have not restricted ourselves to a specific epidemic spread model here because our method is preventive^14^ and can be used before or at the beginning of epidemic for any network. Our strategy for selecting the most appropriate edges to control or remove in the epidemic network is based on reverse link prediction (RLP) explained in Reverse Link Prediction section. The overall process has been depicted in Fig. 4. We are going to search between the current links and find the effective edges with the highest rank to manage or block in order to mitigate the outbreak spread as much as possible before disconnecting the network (step 1). So, we utilize the score functions mentioned with Table 1 in RLP. It is foresighted that controlling or removing the links with more rank from the epidemic network in RLP approach will cause the most delay in epidemic propagation. We will use this algorithm iteratively to construct the new safer network by removing edges with top rank in each repetition; the final predicted network is resulted from removing the edges with the highest TopRanks, in descending order just before the network becomes disconnected by link elimination and stop the possibility of traveling from every node to others (step 2). We will test this idea to find the best score function to be used in RLP. Also, we will test the edge betweenness centrality measure (here after, EB), as a powerful and popular method^28,29^, to compare with our edge removal strategy. Step 2 routine is itself an evaluation measure. Because, the later the method makes the network disconnected, the better is controlling the epidemic with possibility of maintaining the connectivity with more edge removal or control. Finally, we use two extra metrics, total path count and largest Eigenvalue, that will be explained in the next section, to evaluate and select best scoring functions used in RLP (step 3).

To the best of our knowledge, no prior studies have applied link prediction to reduce disease spreading speed.

Of course, our proposed approach is differing from other methods in case of it doesn’t depend on epidemic propagation model and determination of starting points for the infected nodes in the network. Therefore, it will not require simulation and its time complexity and uncertainty as a limiting consequence. Hence, it will be more applicational and easier to understand and implement. However, we will show its superiority on competing method in “Results” section.

First test of the ranking methods for each of the examined networks, is calculating the best edge, and comparing it to randomized version of the edge removal. Similar to other link prediction strategies^30^, in this “null model”, all network nodes have exactly the same connectivity as in the original one, whereas the choice of their edge to remove is totally random. In other words, we should calculate the performance of the random procedure of prioritizing the edges in the same network and compare the results. Fortunately, this examination has been performed before by several references such as^12,14,15,22,24^, and edge betweenness centrality criterion have had superior results than random link removal. Therefore, we do not need to compare our strategy with random deletion of the edges when comparing with the stronger alternative.

### Evaluation criteria

To compare the results of reverse link predictions algorithms we need to use appropriate criteria. According to Table 2, minimizing extreme eigenvalue ($\lambda_{m}$) of the network adjacency matrix is a popular metric. In other words, the extreme eigenvalue of the adjacency matrix of a network demonstrates the speed of infection spreading. This shows that the network connections are more cohesion^13^.

Also, common indicators to compare link removal methods are the largest connected component (LCC) and network efficiency (Eff)^28^. Eff is based on the number of shortest paths, and the LCC represents the number of the nodes in the largest subnetwork of a disconnected network. Researchers use these metrics when removing a fraction of top edges from the network to evaluate the epidemic propagation change. After removing some relations with the highest score, the network may be decomposed into several disconnected parts.

**Figure 5:**
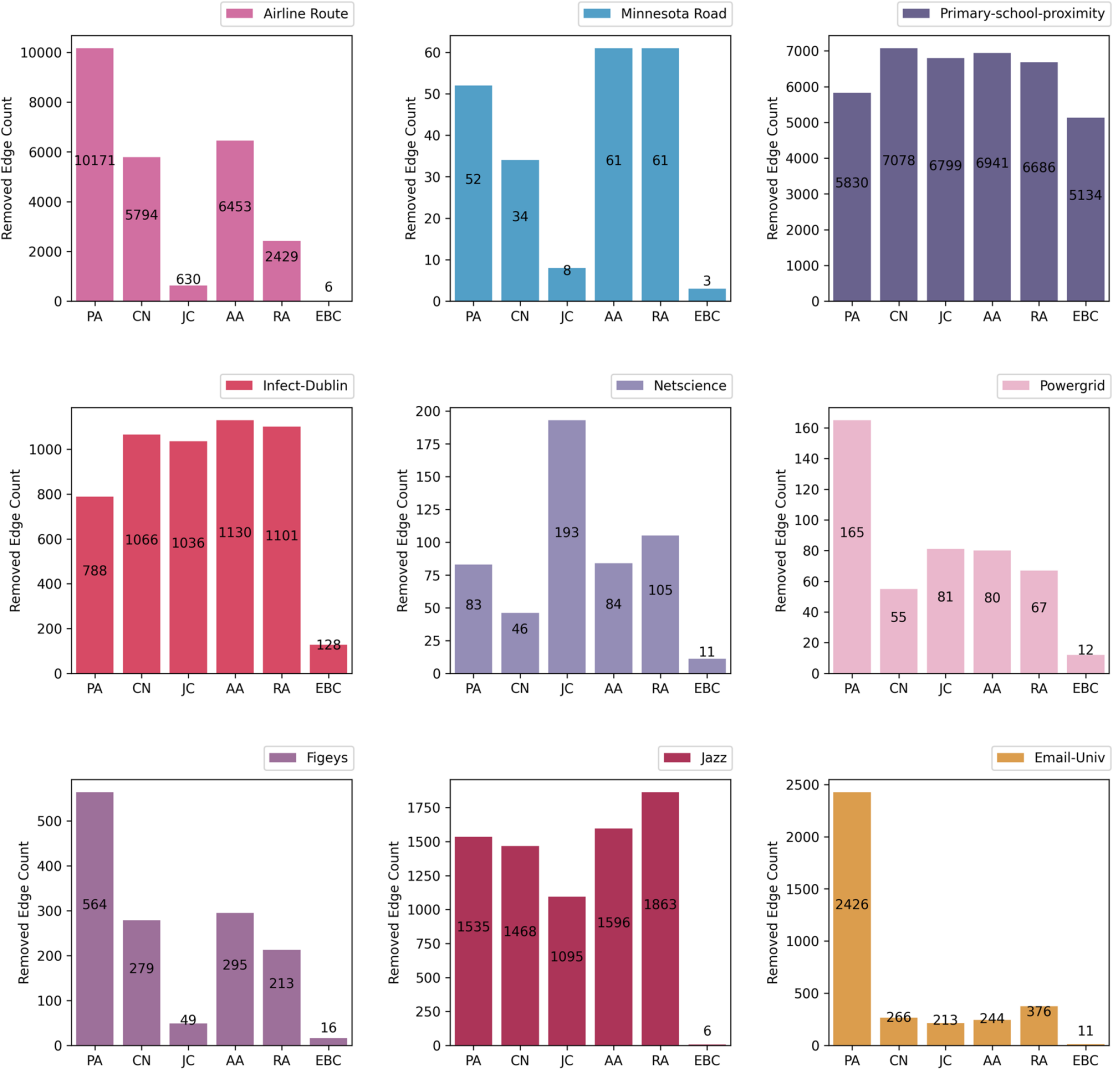
Threshold of disconnectivity over each dataset, maximum removals of edges for various RLP methods plus EB.

However, our approach is focusing on remaining the connectivity of the network after targeted removals. For this reason, LCC is not suitable with our algorithm because we always have a single connected network and continue the removals for each method just before cut off. In other words, an important parameter affecting the assessment is the stop time of controlling or removing the edges. For a transport network it is necessary to remain at least one path between every pair of nodes, cities or persons. So, we will run the EB plus reverse link prediction approach with different score function, AA, CN, JC, RA and PA, to find the threshold of disconnectivity among methods. Disconnectivity threshold for each method simply means a timestep that the method will cut the network into two disjoint parts after several ordered link removals, and disrupts the path availability among the nodes. Minimum threshold between the methods can be utilized as the start time of comparing $\lambda_{m}$ or path count metrics for all the methods because after it, at least one of the methods make the network disconnected and violate the existence of the path between every pair of nodes in the transportation or contact network. Figure 5 depicts the durability of the examined methods to maintain the possibility of communication between the nodes of the network after deleting the links as much as possible. Therefore, the number of removed edges, is offered as a better alternative for LCC in our computations.

For the Eff, it is obvious that not only shortest paths diffuse the epidemic, but also all the paths with different lengths contribute in propagating the epidemics. Instead, we will introduce an easy to understand and more comprehensive indicator enumerating all possible paths in the network. Our metric says that a method is better for epidemic mitigation if it decreases the total number of paths between graph vertices with different lengths more, and therefore decreases the probability of the infection more. Since the number of paths with greater length than 2 is increased proportionally, it is enough to show the number of paths only for a few lengths more than 2 till distinction of its value for different methods.

Therefore, we introduce path count indicator as a new evaluation measure. It is easy to calculate the number of paths between every pair of nodes in the network. Boolean adjacency matrix represents the paths with length 1 between the nodes. Number of paths with at most length *n* between each pair of nodes of a graph can be obtained by multiplying the Boolean adjacency matrix by itself for *n* times.

## Results

In our implementation for each of the five algorithms of link prediction used in RLP plus EB on nine datasets, we reach the connectivity threshold after the number of removals noted in Fig. 5. Normally, EB disconnects all the network quickly because of its nature that tends to find bridge links in the graph. Conversely, PA has the best resistance in cutting off the network for most of the datasets. It means that when we apply PA to a network for decreasing the epidemic, we can control or block more relations than other methods without concern about disconnecting the network. Also, AA and RA perform better for two datasets, and CN and JC has the superior results just for one dataset in cutting the network with more latency. Therefor we select EB values as the maximum number of edge removals to compare the methods in equal conditions, network connectivity. Accordingly, we compare the methods based on largest eigenvalue, $\lambda_{m}$, just before disconnection by the fastest one, EB, Table 4, and if $\lambda_{m}$ could not be distinguished for least number of removals, we delete the relations more until reaching a stable and superior $\lambda_{m}$ for one of the methods. For example, minimum cut number for the Minnesota road dataset is 3 with EB method. However, unique minimum value for $\lambda_{m}$ is not resulted for link prediction scoring functions plus EB method. So, we will remove the edges increasingly and recalculate $\lambda_{m}$ until reaching a unique minimum value for one of the methods. Here the least value for relation removals calculated 8.

**Table 4:** Largest Eigenvalue computed for the network after removing minimum number of links cutting the network*, k*, using reverse link prediction methods and EB*.*

Network	K	PA	CN	JC	AA	RA	EB
Airline route	6	**69.57184**	69.58318	69.84517	69.74546	69.79675	69.84896
Minnesota road	8*	**3.22734**	3.232397	3.232397	3.232396	3.232396	3.22734
Primary-school-proximity	5134	3,377,914	3,061,051	**2,789,986**	2,995,478	2,871,047	4,950,392
Infect-Dublin	788*	**13.888079**	14.250615	15.314465	14.175542	14.007231	13.888079
Netscience	11	9.10295	**8.9833**	9.94036	9.61747	9.61784	10.34319
US power grid	12	7.20656	**6.72104**	7.27246	6.89419	7.31353	7.48305
Human proteins (Figeys)	16	**8.55833**	8.71269	9.15816	8.76006	8.96428	9.1992
Jazz	6	39.65342	**39.65175**	39.92592	39.66885	39.7511	40.00578
Email-Univ	11	**20.25183**	20.42137	20.73929	20.5655	20.67659	20.70212

Star numbers for k column stand for greater value of k because of the inefficient number of edge removals to discriminate the $\lambda_{m}$ for methods.

The best value of $\lambda_{m}$ for each dataset has been bolded.

As introduced before in the evaluation criteria section, counting the number of routes with the length 2 and more will be used as a measure of performance. Number of routes will increase dramatically with the route length. So, with large range of lengths, log values have been used to contain within limited margin for easier comparison. We have supposed maximum path length, the number of travels for each entity, limited to 5 for simplicity. But the method is flexible and can count the path numbers far more lengths (Table 5). Outcomes almost show the superiority of PA method, and are in line with $\lambda_{m}$ evaluation results. However, for Netscience, US power grid, and Jazz datasets, CN ranking receives the best value for $\lambda_{m}$ than PA. For the Primary-school-proximity dataset both path count and largest eigenvalue criteria confirm JC excellence.

**Table 5:** Best method for epidemic mitigation based on two evaluation criteria; total path count and largest eigenvalue.

Network	Path count criteria		$\lambda_{m}$	Largest eigenvalue criteria
	Logarithm of total paths	Superior method		Superior method
Airline route	11.56737	PA	69.57184	PA
Minnesota road	5.25979	PA	3.22734	PA
Primary-school-proximity	34.23164	JC	2,789,986	JC
Infect-Dublin	8.412	PA	13.888079	PA
Netscience	6.44543	PA	7.638144	CN
US power grid	6.2698	PA	6.72104	CN
Human proteins (Figeys)	6.85597	PA	8.55833	PA
Jazz	9.78621	PA	39.65175	CN
Email-Univ	8.87565	PA	20.25183	PA

## Discussion

The most straightforward conclusion of using link prediction techniques in RLP strategy is that there is not a single dominant method for epidemic reduction or control for all networks. This comes back to the nature of the under-test networks and properties of link prediction ranking formulas. Almost all the real-world networks are not essentially regular or completely random, they rather have small-world or scale-free property. While small-world networks have both low shortest path length and high clustering coefficient, scale free networks are known based on their degree distribution tendency to power low property. The small-world networks are similar to regular networks in case of having high clustering coefficient property and are similar to random networks because of their low shortest path lengths. Evidently, in scale-free networks, while most vertices have a low number of connections only, a smaller number of vertices is highly connected. However, it is possible for a network to have both scale-free and small-world properties at the same time^31^.

Besides, PA works fine in scale-free networks, as this ranking formula tend to predict the connotation between high degree not connected nodes, hubs. In comparison, CN, AA and RA behave better in small-world networks that we observe small hop count between every two nodes. So, every two non-connected nodes have few steps far from each other, and normally have neighbors in common with high probability. Of contrast, JC ranking formula is expected to have the best performance in regular like networks, because it normalizes the CN ranking in its calculations.

Accordingly, based on Figs. 2, 3 and Table 3, Airline route, Human proteins and Email-Univ show obvious shape of power law degree distribution. Also, for Minnesota route and Infect-Dublin, average degree is near to 2.5 and 13.5, while most vertices have a lower number of connections than average, and a smaller number of vertices is highly connected (Fig. 2). Likewise, based on Table 3, maximum degree is far more than average degree for both networks. Therefore, the results implicitly show the scale-free property for both Minnesota route and Infect-Dublin networks, and PA ranking has superior results with both path count and largest eigenvalue criteria.

Indeed, scale-free property of the contact networks will cause a small number of highly connected nodes act as hubs that facilitate rapid, near-unstoppable disease spread^14^. Consequently, among the top inter-country or inter-city connections identified by the PA algorithm are the top most busy ones. Similarly, Ref.^22^ confirm that the epidemic threshold can be enhanced by the targeted cutting of links among large-degree nodes for the Barabasi Albert (BA) scale-free networks. PA is a robust method also according to Fig. 5, because it selects the edges that disconnect the network later and gives the more possibility of the operation of the network during epidemic containment in comparison to other methods. In other words, it finds the most redundant links that can be removed from the network with the lowest side effect; for example, minimum restriction for the passengers to go to their destinations in a travel network.

Then for Primary-school-proximity dataset, degree distribution in Figs. 2 and 3, is similar to normal. Here, average clustering coefficient is approximately high, and low value for network diameter and average shortest path length demonstrate the balanced degree distribution between nodes. These properties confirm the superiority of JC performance for the network. Despite these, Netscience, US power grid and Jazz datasets, have PA as the best ranking formula for path count criteria, and CN as the foremost performance based on $\lambda_{m}$. Clearly, the difference between the largest eigenvalue for CN and PA is very low for these datasets and PA is the nearest value to CN (Table 4). Netscince and Jazz presents high clustering coefficient with low average path length. The only contradicting results is with US Power grid network that is a sparse network and tends to be more power law based on its degree distribution charts in Figs. 2 and 3. But, it shows small-world attribute at the same time based on the $\lambda_{m}$ result.

The overall results show that RLP works better with PA algorithm according to the path count and $\lambda_{m}$ measures, even though the largest eigenvalue is temporarily greater for CN than PA for some datasets (Tables 4, 5). Edge betweenness centrality as a basic algorithm, has the worst results in controlling the epidemic spread in comparison with the link prediction algorithms.

Our approach is model free because it is preventive and finds the critical edges, before any epidemic event. It also does not need to specify start nodes of infection; i.e., any node, person, city or airport, can be an initial node for the outbreak. List of links computed with the strategy can identify which connections of each node should be controlled sooner. These properties will make the approach more general and effective than those limited to a specific epidemic model or limited start points. Of course, our approach is not essentially superior to other epidemic speed reduction methods. The main reason is that according to Table 2, it is not easily possible to compare the methods because of their different specifications and conditions. But, RLP is general and easy to understand and compute. According to our review and investigations, only the JC, from the link prediction algorithms, has been used before^12^. RLP strategy also does not require simulating the propagation of the disease in the network in a stochastic manner and running a simulation program several times, because the method can prioritize the edges in only one run. It does not need to provide a fair way to select the starting nodes of the pandemic also, Owing to its consideration of identical metric for all the nodes and connections between them.

It is worth to note that the difference between our research and network robustness analysis is that the primary assumption of our method is to retaining the connectivity of the network as much as possible, and our process stops with first disconnectivity of the network. Because we are aiming at preserving the connection between all nodes, such as flight relations in an aviation network, with maximal restriction of the epidemic’s propagation. While in network robustness that is mainly based on node removal, the primary goal is to limit malfunctioning of one or more nodes in a network^32^. Even though, there are cases that include link failure as robustness problem in the network^33^. Here, testing the performance of the solutions is not dependent on preserving the connectivity of the modeling graph. Most recent edge-based robustness analysis papers are Refs.^28,29^ that we compared our method with their best reported indicator, edge betweenness centrality, in this research accordingly.

## Conclusion

Current link prediction strategies only consider the forward approaches in order to add new probable links to the network or remove the weak ones from. Another different view to link prediction is identification of the importance of the available links; finding the newly established relations with reverse link prediction strategy as a method of edge prioritization. In this research, we implemented the new strategy for link prediction, RLP, and used node neighborhood similarity-based algorithms as its core to find and prioritized the most important relations in contact networks with different levels.

A successful and evident case for RLP application is epidemic mitigation. It has been proved that international air travel restrictions may provide important delay in the spread of a pandemic^34^ specially when combining with other transportation methods. But this can be very time and budget consuming regard to the huge size of the network. Fast and efficient topology-based link prediction methods proposed here, can prioritize the transport relations between cities and places, in order to help the health and governmental organizations to react to pandemics as soon as they appear in a region. Our approach gives the list of edges in decreasing priority sequence to define the right order of control.

Besides, there exists numerous link prediction algorithms including path based and supervised methods^7^ to improve the current results. The investigation of these options is an agenda for future work. Similar to the node neighborhood link prediction methods, other ranking formulas may be investigated based on their properties to help choose best appropriate methods based on the examined network.

RLP can be also used with other types of networks like weighted or directed ones for better modeling. For example, the strength of a connection between two airports can be measured by the number of flights or passenger capacity, i.e., the number of passengers that travel a given route per day can be more meaningful. Taking the weight of the edges into account will probably improve the outcomes as there are the weighted versions of link prediction to achieve better results^35,36^. Evidently, some epidemic control solutions have been proposed to work with weighted networks also^37^.

An interesting future development of RLP may be expanding it to multilayer networks. Transport networks are not only restricted to aviation only. Road, railway, water and air transport networks can be used with the method to predict removing or controlling relations in order of their priority. An integration of all the transport types will establish a multilayer network. Nevertheless, preparing and combining the data for such network is not easily possible. However, the method can be used as an optimization solution, based on a specific set or a number of edges that can be canceled or controlled. We can even use RLP when the epidemic starts, reactive mode, restricting the method to find the most critical connections between infected and susceptible nodes. For instance, if we are going to separate the infected nodes from susceptible ones in a SI epidemic model, we may only consider the edges that are currently between infected and susceptible nodes.

## Data Availability

The datasets analysed during the current study are available at: https://networkrepository.com/primary-school-proximity.php; https://networkrepository.com/infect-dublin.php; https://openflights.org/data.html#route; https://networkrepository.com/road-minnesota.php; http://konect.cc/networks/maayan-figeys/; https://networkrepository.com/email-univ.php; https://networkrepository.com/netscience.php; http://konect.cc/networks/arenas-jazz/; http://konect.cc/networks/opsahl-powergrid.
